# Development of a three-dimensional organoid model to explore early retinal phenotypes associated with Alzheimer’s disease

**DOI:** 10.1038/s41598-023-40382-4

**Published:** 2023-08-24

**Authors:** Sailee S. Lavekar, Jade Harkin, Melody Hernandez, Cátia Gomes, Shruti Patil, Kang-Chieh Huang, Shweta S. Puntambekar, Bruce T. Lamb, Jason S. Meyer

**Affiliations:** 1 Department of Biology, Indiana University-Purdue University Indianapolis, Indianapolis, IN 46202 USA; 2 Stark Neurosciences Research Institute, Indiana University School of Medicine, Indianapolis, IN 46202 USA; 3 Department of Pharmacology and Toxicology, Indiana University School of Medicine, Indianapolis, IN 46202 USA; 4 Department of Medical and Molecular Genetics, Indiana University School of Medicine, Indianapolis, IN 46202 USA; 5 Department of Ophthalmology, Glick Eye Institute, Indiana University School of Medicine, Indianapolis, IN 46202 USA

**Keywords:** Cellular neuroscience, Induced pluripotent stem cells

## Abstract

Alzheimer’s disease (AD) is a progressive neurodegenerative disorder characterized by the accumulation of Aβ plaques and neurofibrillary tangles, resulting in synaptic loss and neurodegeneration. The retina is an extension of the central nervous system within the eye, sharing many structural similarities with the brain, and previous studies have observed AD-related phenotypes within the retina. Three-dimensional retinal organoids differentiated from human pluripotent stem cells (hPSCs) can effectively model some of the earliest manifestations of disease states, yet early AD-associated phenotypes have not yet been examined. Thus, the current study focused upon the differentiation of hPSCs into retinal organoids for the analysis of early AD-associated alterations. Results demonstrated the robust differentiation of retinal organoids from both familial AD and unaffected control cell lines, with familial AD retinal organoids exhibiting a significant increase in the Aβ42:Aβ40 ratio as well as phosphorylated Tau protein, characteristic of AD pathology. Further, transcriptional analyses demonstrated the differential expression of many genes and cellular pathways, including those associated with synaptic dysfunction. Taken together, the current study demonstrates the ability of retinal organoids to serve as a powerful model for the identification of some of the earliest retinal alterations associated with AD.

## Introduction

Alzheimer’s disease (AD) is the most common form of dementia, currently affecting more than 6 million individuals in the United States alone^1,2^. It is primarily characterized by the accumulation of extracellular plaques of amyloid-β (Aβ) and the hyperphosphorylation of microtubule associated protein tau, resulting in intraneuronal neurofibrillary tangles within the brain, leading to the degeneration of neurons^3,4^. Numerous underlying factors may contribute to the onset of AD, including genetic factors that may cause or contribute to the progression of the disease state^5,6^. However, studies have shown that neuropathological changes in AD begin decades before the clinical onset of cognitive decline^7,8^. Similarly, as earlier diagnoses allow for a greater opportunity for therapeutic intervention, a critical need exists for the development of approaches to screen for the earliest detectable changes associated with AD progression^9,10^.

The neural retina is an extension of the central nervous system within the eye that can be readily visualized through non-invasive ophthalmic imaging examinations^11–13^. Previous studies have demonstrated the onset of AD-related phenotypes within retinal tissue^14–17^, perhaps even earlier than the onset of phenotypes within the brain, highlighting the potential to use AD-associated retinal changes as predictors of disease progression in the central nervous system, and to develop earlier strategies for therapeutic intervention^18–21^. Despite this potential, relatively little is known about the retinal phenotypes that may be detected at the earliest stages of the disease process^22^, which would provide greater opportunities for therapeutic intervention. As such, a critical need exists for the development of novel tools for the identification of early retinal AD phenotypes that may eventually be developed as potent biomarkers for the earliest stages of the disease state.

To address this shortcoming, the current study focused on the development of human pluripotent stem cell (hPSC)-derived retinal organoids as a tool to model the earliest stages of retinal AD phenotypes^23^. Retinal organoids closely mimic the development and three-dimensional organization of the retina^24–30^, resulting in stratified optic cup-like structures that effectively represent early stages of retinal tissue. Given these features, the potential exists for retinal organoids to be further analyzed for AD-related phenotypes as biomarkers at stages long before the onset of observable symptoms in patients. To accomplish this, we leveraged two existing lines of patient-derived human induced pluripotent stem cells with familial forms of AD^31^, with disease-causing gene variants in PSEN1 (A246E) and PSEN2 (N141I), as well as two unaffected control cell lines for the differentiation of retinal organoids and subsequent analysis of AD phenotypes. Retinal organoids were effectively differentiated from all cell lines and subsequently, hallmark phenotypes of AD were identified in AD retinal organoids, including an increase in the Aβ42:Aβ40 ratio as well as an increase in hyperphosphorylated Tau (pTau). Given the identification of characteristic AD phenotypes, we then sought to explore the identification of additional disease-associated phenotypes. Nanostring transcriptional profiling identified a number of differentially expressed genes (DEGs) between AD and unaffected control cell lines, including changes in cellular pathways suggesting synaptic dysfunction within AD retinal organoids. Taken together, the results of this study demonstrate the feasibility to use retinal organoids as a powerful tool for the analysis and identification of early retinal AD phenotypes, which may then be used to eventually translate these findings into approaches for the early identification of disease-related phenotypes in AD patients, allowing for early intervention.

## Results

### Validation of pluripotent stem cells with AD-related gene variants and associated controls

**Figure 1:**
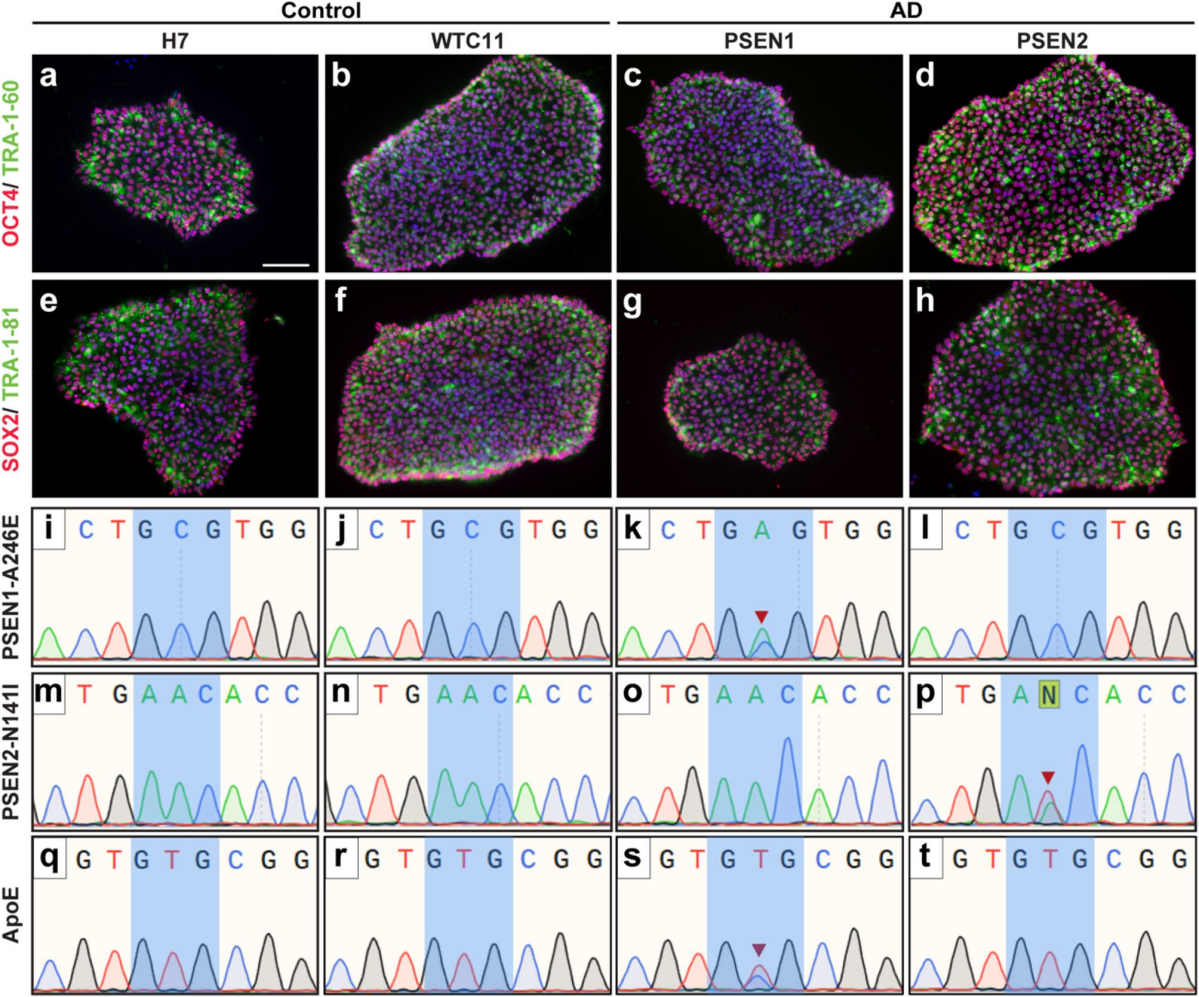
Confirmation of pluripotency and presence of AD-associated gene variants in human pluripotent stem cells used in this study**.** (**a**)–(**h**) Two control cell lines (H7 and WTC11) and two lines of AD patient-derived iPSCs (PSEN1 and PSEN2) were validated for pluripotency via immunocytochemistry to confirm expression of cell surface markers such as Tra-1-60 and Tra-1-81 and the transcription factors OCT4 and SOX2. Nuclei are counterstained with DAPI in each image in blue. (**i**)–(**t**) Validation of the presence or absence of the respective AD associated SNPs by Sanger sequencing (PSEN1-A246E, PSEN2-N141I, ApoE). Red arrowhead indicates presence of heterozygous SNP. All four cell lines were used in these experiments, and immunostaining was performed on at least 3 independent passages of each cell line. Scale bar equals 100 um.

To effectively explore the ability of hPSC-derived retinal organoids to serve as an effective in vitro of AD retinal pathologies, we selected two cell lines with familial AD SNPs (PSEN1-A246E and PSEN2-N141I), as well as two unaffected control cell lines (H7 and WTC11). Initially, the pluripotency of these cell lines was validated using antibodies against putative pluripotency markers including the OCT4 and SOX2 transcription factors as well as the Tra-1-60 and Tra-1-81 cell surface markers using immunocytochemistry (Fig. 1a–h), demonstrating the pluripotent nature of these cell lines. Further, Sanger sequencing confirmed the presence or absence of the identified AD-related SNPs (Fig. 1i–p), with appropriate SNPs found exclusively within the expected cell lines. As many gene variants can be associated with AD, and variants in the ApoE gene confer high risk for AD, we also screened these cell lines for and ApoE3 or ApoE4 genotype (Fig. 1q–t), demonstrating that the PSEN1 AD cell line also possessed an ApoE3/4 genotype conferring increased risk for AD, while the other cell lines maintained a neutral ApoE3/3 genotype. Taken together, these results demonstrate the pluripotency of the cell lines used within this study, as well as that these cell lines express relevant AD-specific mutations, allowing for the pursuit of the subsequent goals of this study, namely the derivation of retinal organoids for the identification of early AD-related phenotypes.

### Efficient derivation of retinal organoids from both AD and control hPSCs

**Figure 2:**
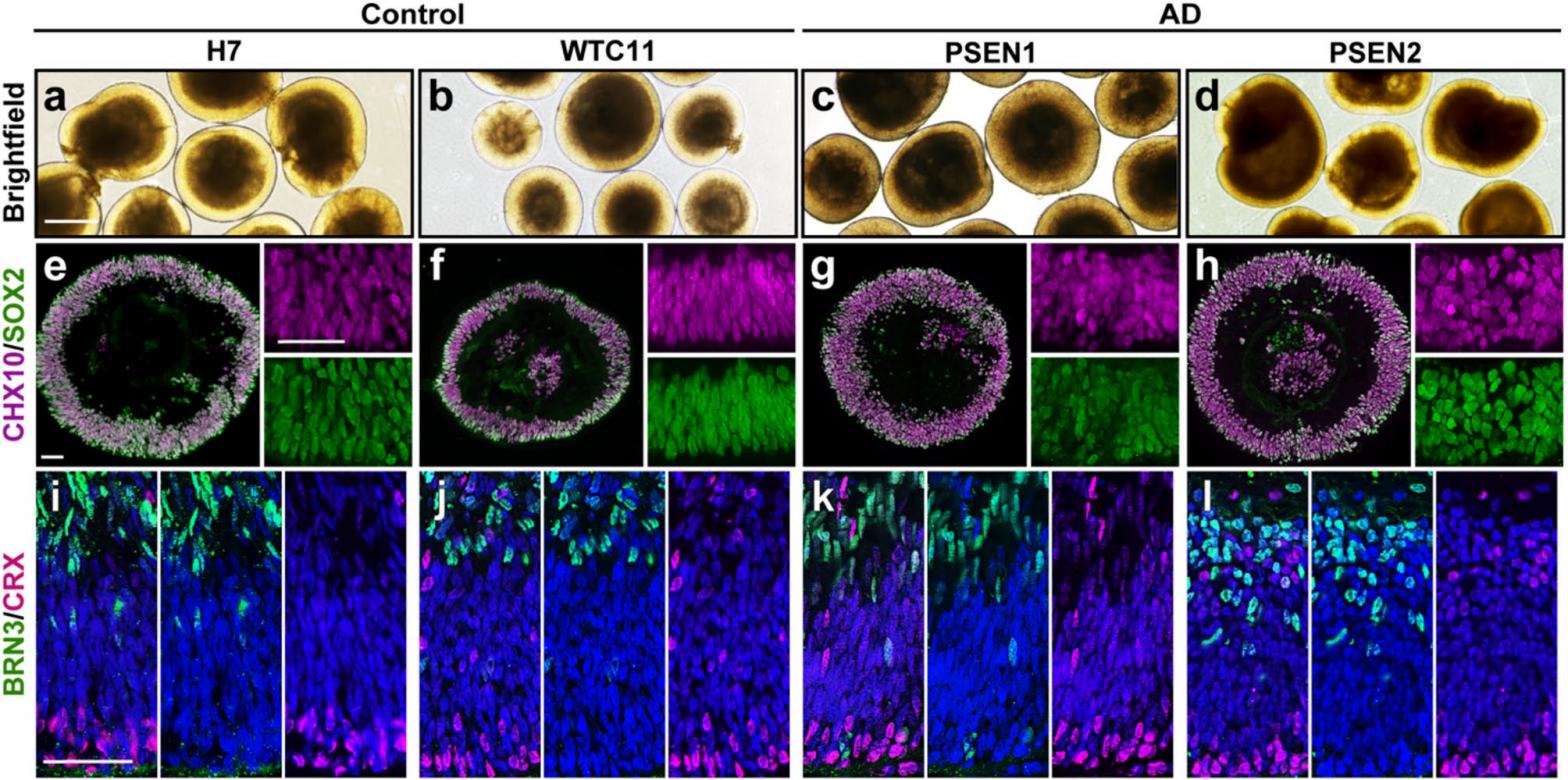
Efficient differentiation of stratified three-dimensional retinal organoids from both AD and control cell lines. (**a**)–(**d**) Retinal organoids derived from control and AD cell lines exhibited characteristic morphological features including a stratified retinal neuroepithelial layer by brightfield microscopy. (**e**)–(**h**) All cell lines were capable of generating early, optic vesicle-like retinal organoids after a total of 30 days of differentiation that were highly enriched for retinal progenitor markers including CHX10 (magenta) and SOX2 (green). (**i**)–(**l**) Further differentiation of retinal organoids from each cell line to day 70 of differentiation revealed the stratification of differentiated retinal cell types, including the proper organization of retinal ganglion cell layers on the inner layers identified by BRN3 expression (green) and photoreceptors located along the outer layers identified by CRX expression (magenta). In (**e**)–(**l**), all nuclei are counterstained in blue with DAPI. All four cell lines were used in these experiments, and immunostaining of retinal organoid sections was performed on a minimum of at least 3 unique differentiation experiments from each cell line. Scale bars equal 500 µm for (**a**)–(**d**), and 50 µm for (**e**)–(**l**).

Next, we sought to determine if AD and control lines of hPSCs could effectively yield retinal organoids following established differentiation protocols^24,28,32–38^. Initially, we observed that retinal organoids could be effectively derived from hPSCs from both AD as well as control cell lines, based upon morphological features including a golden, stratified appearance around the outer layers of the organoids (Fig. 2a–d). We then sought to determine if retinal organoids differentiated from each of these cell lines differentiated through both early and later stages of differentiation. After 30 days of differentiation, each cell line yielded optic vesicle-like early retinal organoids that were highly enriched for retinal progenitor cell markers, including CHX10 and SOX2 (Fig. 2e–h). Further differentiation of these primitive retinal organoids allowed for the further maturation and stratification of differentiated cell types, such as retinal ganglion cells within the inner layer of organoids as well as photoreceptors found lining the outer layers of the organoids, with this expression pattern similar across all control and AD lines (Fig. 2i–l). Collectively, these results demonstrated the ability to yield stratified retinal organoids with a spatial and temporal patterning that was similar across AD and control cell lines, further justifying the exploration of AD-related phenotypes within these organoids.

### Assessment of AD-related phenotypes within retinal organoids

**Figure 3:**
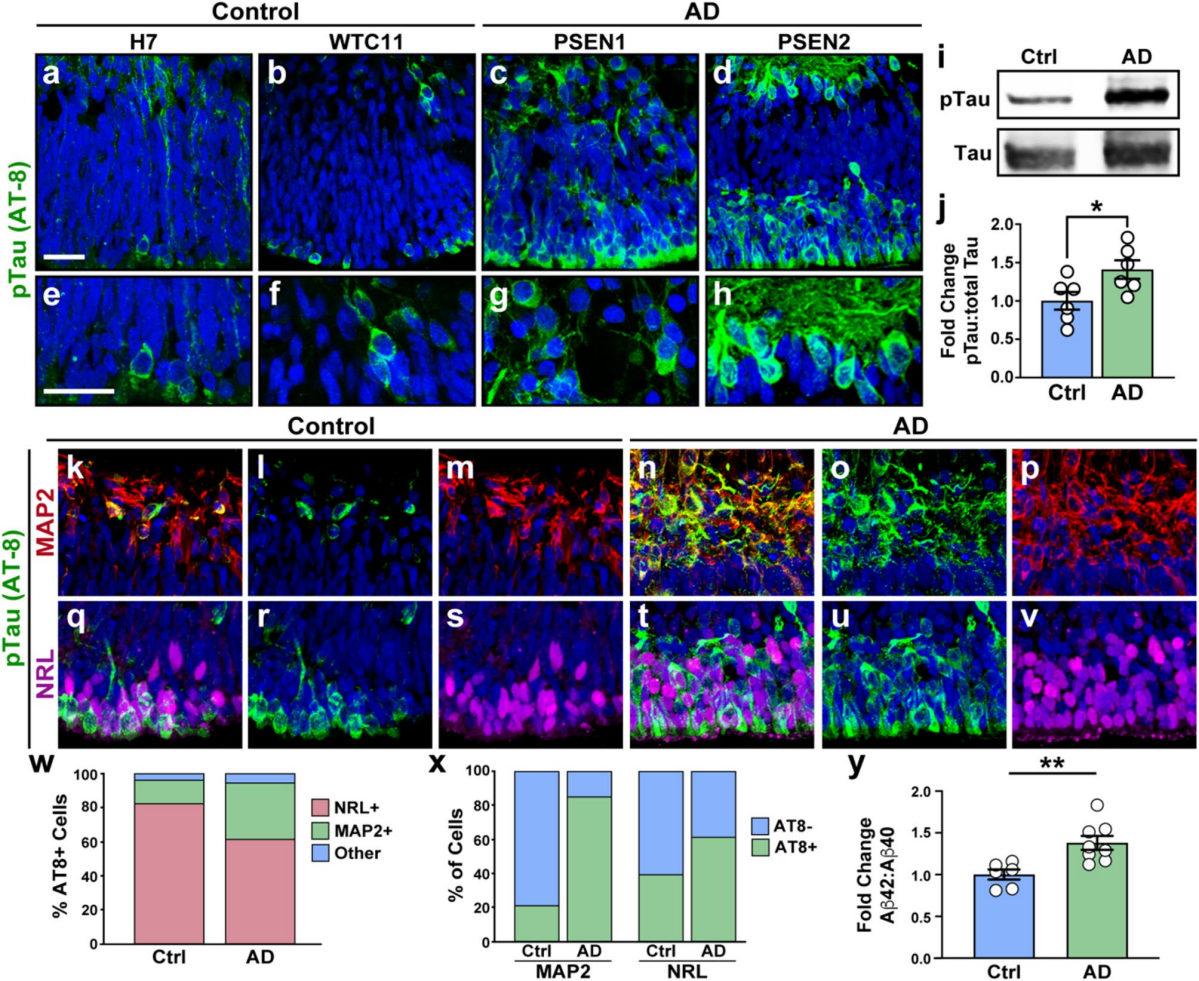
Identification of AD-related pathological features within retinal organoids. (**a**)–(**h**) pTau expression (AT-8, green) was observed in all retinal organoids after 150 days of differentiation, with levels increased within AD retinal organoids with either PSEN1 or PSEN2 SNPs. Panels a-d demonstrate lower magnification representing all layers of retinal organoids, while panels (**e**)–(**h**) are higher magnification to visualize greater detail of pTau-expressing cells. (**i**)–(**j**) Western blot analyses of retinal organoid samples demonstrated a significant increase in pTau (AT-8) expression within AD retinal organoids. (**k**) Similarly, an increase in the Aβ42:Aβ40 ratio was observed in the conditioned medium of AD retinal organoids after 5 months of differentiation as observed by ELISA assays. Data in i-y was obtained from the H7 and PSEN1 cell lines and is representative of results obtained across all cell lines. Immunostaining was performed on a minimum of 5 independent differentiation experiments per cell line. Western blot results are from a total of 18 differentiation experiments (H7 n = 6, WTC11 n = 3, PSEN1 n = 6, PSEN2 n = 3). Measurements of Ab are from a total of 23 differentiation experiments (H7 n-6, WTC11 n = 5, PSEN1 n = 8, PSEN2 n = 4). Nuclei in all microscopy images were counterstained with DAPI in blue. Statistical analyses were performed via students *t*-test with a *p* < 0.05 to determine significance. Scale bars equal 25 μm for all images.

Increases in the levels of phosphorylated Tau protein (pTau), as well as an increase in the relative abundance of Aβ42 fragments, are hallmarks of AD pathology in the brain, and similar phenotypes have also been observed in the retinas of AD patients^39,40^. While our results have demonstrated the ability to effectively derive retinal organoids from both AD and control cell lines, it was unclear whether these hallmark features of AD pathology could be observed in this in vitro cellular model. Thus, we analyzed retinal organoids after several months of differentiation for changes in the expression of both pTau (AT-8) as well as the Aβ42:Aβ40 ratio^41,42^. Within 5 months of differentiation, we observed an increase in the relative abundance of pTau (AT-8) in AD retinal organoids compared to control cell lines (Fig. 3a-h), as well as a significant increase in the ratio of pTau:total Tau by western blotting (Fig. 3i-j, Supplemental Fig. 1). Next, since the expression of pTau seemed to be localized primarily in the innermost and outermost layers of the retinal organoids, we explored whether or not this expression indicated cell type specificity for pTau. Indeed, we observed that the large majority of pTau-positive cells co-localized with either MAP2 (RGCs) or NRL (photoreceptors) at 5 months of differentiation (Fig. 3k–w). Interestingly, while pTau immunoreactivity was found most abundantly in the outer regions of the retinal organoids (co-localized with NRL), we observed the largest increase in pTau expression between control and AD organoids in the inner regions of the organoids (co-localized with MAP2, Fig. 3x), suggesting that RGCs are primarily affected with these AD mutations. Finally, across control and AD organoids, we also observed a significant increase in the Aβ42:Aβ40 ratio in the conditioned media as measured by ELISA analyses (Fig. 3y) at 5 months of differentiation. Taken together, these results demonstrated that hallmark characteristics of AD pathology, including those that have previously been demonstrated within the retina of AD patients, can also be effectively recapitulated in AD retinal organoids, further validating this in vitro model for further studies of AD-related phenotypes.

### Identification of transcriptional profiles associated with AD within retinal organoids

**Figure 4:**
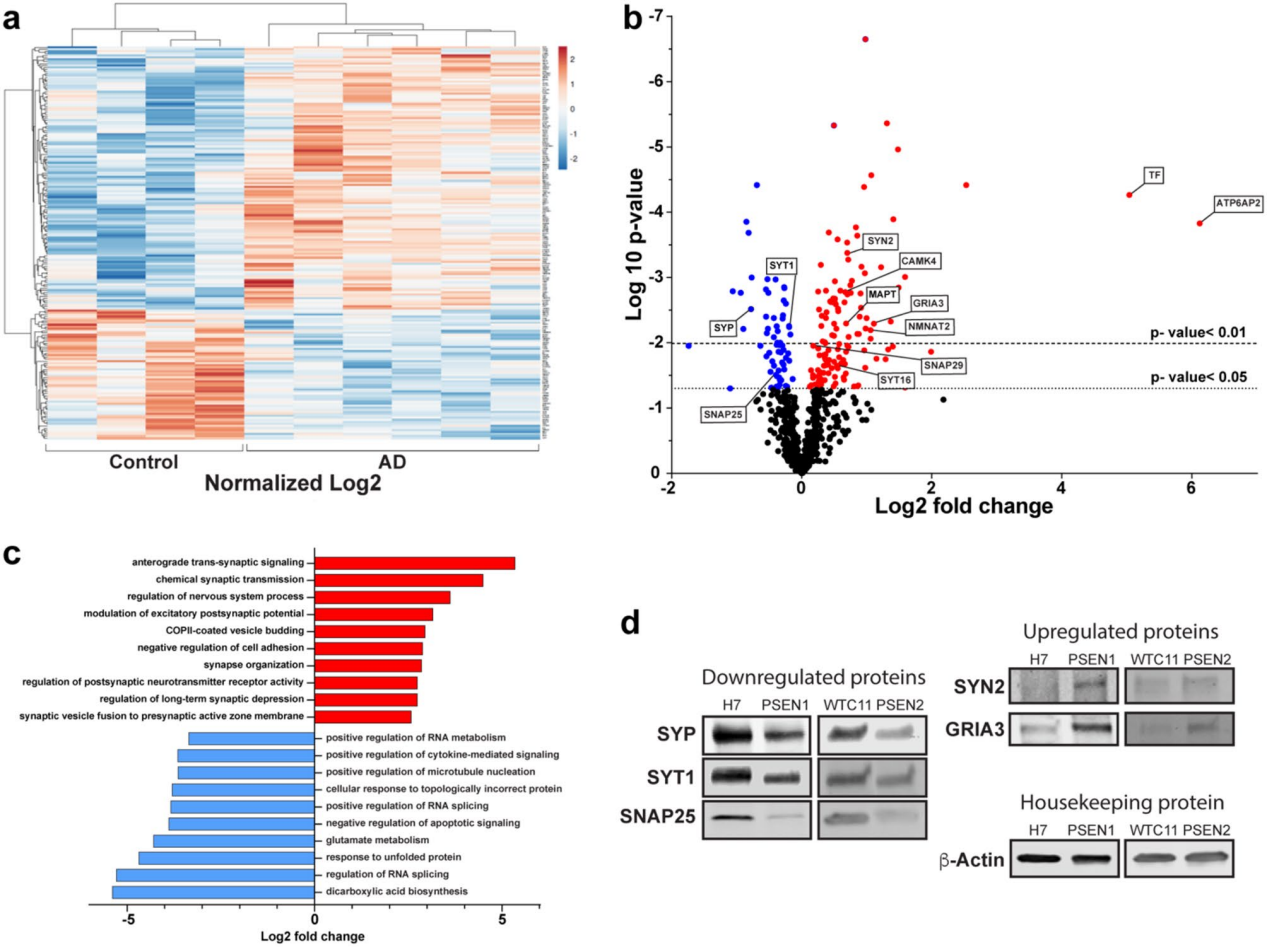
Transcriptional profiling of AD retinal organoids suggests synaptic dysfunction as a characteristic feature. (**a**) Transcriptional profiling was performed using the Nanostring nCounter human AD panel, and a heatmap was generated to compare differentially expressed genes (DEGs) between AD and control retinal organoids at 150 days of differentiation. (**b**) Volcano plot illustrating genes that were significantly up- or down-regulated, with candidate genes highlighted. (**c**) Pathway analysis based upon differential gene expression illustrating cellular pathways that were modulated in AD retinal organoids, in particular pathways associated with synaptic dysfunction. (**d**) Western blot analyses validated transcriptional differences associated with synaptic dysfunction in AD retinal organoids. Transcriptional profiling via Nanostring analyses was performed with the H7 and PSEN1 cell lines using 4 samples from H7 and 6 samples from PSEN1 cell lines, while western blot confirmed protein expression using all four cell lines from at least 3 independent differentiation experiments.

Our initial results demonstrated the feasibility of assessing AD-related phenotypes within patient-derived retinal organoids, despite the fact that retinal organoids still represent early stages of retinogenesis. To further investigate pathological changes that may be identified at these earliest stages of disease, we conducted transcriptional analyses using the Nanostring human Alzheimer’s Disease gene panel to identify differentially expressed genes (DEGs) and cellular pathways between AD and control retinal organoids (Fig. 4, Supplemental Table 2). Significant transcriptional differences were observed between the control and AD-derived retinal organoids, with samples properly segregating based upon their transcriptional profiles (Fig. 4a). Among the differentially expressed genes, 130 upregulated genes and 64 downregulated genes were identified (Fig. 4b), and pathway analysis demonstrated the differential regulation of several cellular pathways (Fig. 4c). Interestingly, many differentially expressed genes and pathways were associated with synaptic plasticity and dysfunction. To further validate that the changes observed at the transcriptional level were then recapitulated at the protein level, we performed Western blot analyses that confirmed the differential regulation of proteins associated with synaptic function (Fig. 4d, Supplemental Fig. 2). Taken together, these results help to demonstrate the suitability of hPSC-derived retinal organoids for the investigation of early retinal phenotypes associated with AD.

## Discussion

The results described within this paper demonstrate the ability to direct the differentiation of retinal organoids from human pluripotent stem cells, particularly those with SNPs related to familial forms of Alzheimer’s Disease (AD). In these studies, AD retinal organoids faithfully recapitulated pathological hallmarks of the disease state, including an increase in Aβ42:Aβ40 ratio as well as an increase in the level of phosphorylated tau. While the retina has often been referred to as a window into the central nervous system due to the ease of access to and.

visualization of the neural retina within through the lens of the eye^22^, our knowledge of AD-related phenotypes is largely limited to later stages of the disease when donor retinal tissue is commonly studied^43^. Thus, the data provided within this study demonstrates that it may be possible to identify early, disease-associated cellular changes in the pre-clinical stages of AD. As it is known that AD-related changes can manifest even decades before a clinical diagnosis^44^, the ability to identify early disease-associated changes would facilitate an earlier diagnosis and enable greater opportunities for intervention. Given that retinal organoids represent the earliest stages of human retinal development and by association, the earliest stages of disease states, our results demonstrate the feasibility of identifying early AD-related changes that may be further developed as clinical indications of the earliest changes in the disease state.

Within this paper, we utilized two unrelated lines of iPSCs derived from patients with familial forms of AD (PSEN1 and PSEN2 gene variants), as well as two unaffected control stem cell lines. Regardless of the genotype of these cell lines, each were similarly capable of yielding retinal organoids that mimicked the early optic vesicle state of retinal development as characterized by robust expression of CHX10 and SOX2, followed by the further differentiation and stratification of retinal cell types, including retinal ganglion cells (RGCs) on basal layers of the organoids as well as photoreceptors localized along the apical edges. While no disease related phenotypes were observed at early stages of organoid differentiation, we found that retinal organoids with familial AD gene variants efficiently recapitulated classical AD-associated phenotypes within 5 months of differentiation, including increased levels of pTau as well as an increase in the Aβ42:Aβ40 ratio. Importantly, these results were reproducible across two independent lines of patient-derived cells compared to two independent control cell lines. Interestingly, while the most profound phenotypes were observed at the latest timepoint analyzed within this study at 5 months of differentiation, we were able to observe subtle changes in these phenotypes associated with the AD cell lines as early as 3 months of differentiation, suggesting that cellular changes associated with the disease state may manifest very early in the retina. Indeed, we observed a robust increase in the expression of pTau in the AD organoids at these early stages. Previous studies have indeed demonstrated an increase in pTau within the retina of AD models, mostly within the retinal ganglion cells and the optic nerve as the connection between the eye and the brain^45–47^, associated with some features of AD pathology and visual dysfunction. A few other studies have also observed pTau in other retinal neuronal populations, although this increase in pTau in other retinal neurons has not been clearly tied to functional deficits^48,49^. Similarly, our results show a similar pattern of distribution of pTau, mostly in RGCs and photoreceptors. In retinal organoids from control cell lines, most of the observed pTau expression was found associated with photoreceptors in the outer layers, with these levels increasing somewhat in AD lines. However, while there was sparse pTau expression associated with RGCs in control retinal organoids, there was a profound increase in expression in the AD lines, mirroring what has been seen in the RGC layer and nerve fiber layer in previous studies^46^. Further, the increase in the Aβ42:Aβ40 ratio mimics what has been previously observed in the brain, as well as other stem cell models of AD^50,51^. While as a biomarker, the levels of Aβ42:Aβ40 in CSF are known to decrease in AD^52,53^, the pathological Aβ42 form is predominantly found in depositions within the brain. The increased Aβ42:Aβ40 ratio observed extracellularly in our results is likely analogous to the release of Aβ within brain or retinal tissue.

Given the ability to identify hallmark features of AD within retinal organoids, we then investigated the ability to identify additional pathological changes associated with the disease state by transcriptional profiling of retinal organoids after 5 months of differentiation. It was notable that we observed changes in the expression levels of genes related to synaptic connectivity, in line with studies suggesting that a loss of synaptic connectivity may also be an early indicator of AD pathology that occurs before the onset of plaque deposition^54,55^. While previous studies have demonstrated synaptic dysregulation in AD^56–58^, there are limited analyses of synaptic dysfunction in the retina associated with AD. Thus, these studies represent an important direction for early phenotypes within the retina that may aid in the identification of AD phenotypes observed before clinical phenotypes manifest, aiding in future translational applications that could help to better understand how preclinical alterations in AD affect disease progression. Importantly, it should be noted that while the transcriptional identification of synaptic dysregulation as well as validation via western blots provides intriguing directions for future investigation, the expression of synaptic transcripts and even proteins is not definitive evidence for the existence of functional synapses, much less rearrangement of functional synapses. Future studies will need to explore these phenotypes in closer detail. Nevertheless, the results of these experiments underscore the potential of retinal organoids for the identification of future retinal biomarkers for AD, as well as in the identification of candidate pathways for earlier therapeutic intervention^59^.

While the results of this study establish retinal organoids as an in vitro model for the identification of early retinal phenotypes associated with AD, there are also many opportunities for further investigation. Importantly, any findings associated with in vitro retinal organoid studies need to be validated in tissue obtained from patient sources to ensure these phenotypes are recapitulated within the eye. Additionally, differences between retinal organoids from batch to batch can introduce variability between experiments, complicating interpretations of some results. Improvements to retinal organoid differentiation protocols to yield more consistent organoids could help solve this issue, as would the use of gene edited isogenic pairs of disease and control cell lines. In this context, previous studies have utilized isogenic pairs of cells to minimize variability due to differences between individuals, reducing the number of cell lines needed for investigation^37,50,60^. Further, the lack of immune cells such as microglia in these retinal organoid systems can also be an important future direction to address, as studies in recent years have underscored a pivotal role for microglia in the regulation and progression of AD^51,61–63^, including neuronal vulnerability to the development of neurofibrillary tangle pathology and subsequent neurodegenerative outcomes. With recent advances in the differentiation of iPS cell-derived microglial-like cells^60,64,65^, future studies will be able to assess the role of microglia in retinal organoids by the addition of hPSC derived microglia to better understand their contribution and disease etiology in AD, as has been recently described in other systems^65,66^. Finally, as previous studies have suggested that AD phenotypes manifest in the retina earlier than those within the brain^18–21^, future studies will allow for a more in-depth assessment of these changes through a comparison of retinal and brain organoids, including an analysis of those changes in common among both populations, as well as the timing of onset for these changes.

Taken together, the results of this study demonstrate the ability to use human pluripotent stem cell-derived retinal organoids as a powerful in vitro model for the identification of some of the earliest pathological changes associated with AD. Based upon these approaches, many new and exciting opportunities now exist for the identification and analysis of early retinal changes associated with AD to gain a better understanding of how these early changes affect neuronal health and function. Further, these results highlight the potential that AD retinal organoids may be further developed as tools for the establishment of possible diagnostic approaches for the assessment of disease pathology, long before the onset of clinical symptoms, and have the potential to serve as a platform for the high-throughput screening of pharmacological compounds for their efficacy.

## Methods

### Maintenance and expansion of human pluripotent stem cells

Four different lines of human pluripotent stem cells were used in this study, including two patient-derived cell lines with familial Alzheimer’s Disease gene variants (PSEN1-A246E and PSEN2-N141I), as well as two unaffected control cell lines (H7 and WTC11)^67,68^. Both AD lines as well as the WTC11 cell line were obtained from the Coriell Repository (catalog ID AG25367, AG25370, and GM25256, respectively), while the H7 (WA07) cell line was previously obtained from WiCell. These cell lines represent both an embryonic stem cell line (H7) as well as iPS cell lines (WTC11, derived from leg skin fibroblast; both PSEN lines, derived from arm skin fibroblasts). All cell lines were grown and expanded as previously described^30,32,33,69^. Briefly, cells were maintained in the undifferentiated state using mTeSR1 medium and grown on either a Matrigel or Geltrex substrate. Cells were passaged when they reached between 70 and 80% confluency using dispase (2 mg/ml) and split at a 1:6 ratio, approximately every 5–6 days.

### Differentiation of retinal organoids

hPSCs were differentiated into retinal organoids using a stepwise differentiation protocol as previously described^32,33,37,38^. Upon reaching 80% confluency, colonies of hPSCs were enzymatically lifted from the plates using dispase (2 mg/ml) and grown in suspension culture to allow the formation of embryoid bodies (EBs). EBs were then transitioned from mTeSR1 to Neural Induction Medium (NIM; consisting of DMEM/F12, 1% N2 supplement, 1% MEM non-essential amino acids, 1% anti-anti, and heparin (2 mg/ml)) over the first 3 days of differentiation. To induce retinal differentiation, BMP4 (50 ng/ml) was added to cultures on day 6 of differentiation. EBs were then induced to adhere to the culture plates with the addition of 10% fetal bovine serum on day 8 in NIM, with the gradual reduction of BMP4 by half media changes on days 9 and 12. After 16 days of differentiation, retinal organoids were detached from the plate and maintained in Retinal Differentiation Medium (RDM; DMEM/F12 (3:1) supplemented with 2% B27 supplement, 1% MEM non-essential amino acids, and 1% anti-anti), with media changes every 2–3 days. Cultures were further supplemented with FBS in a stepwise manner to 10% final concentration starting at day 16. Cultures were further supplemented by the addition of 100 µM taurine after 30 days of differentiation and 1 µM of retinoic acid from 60 to 90 days of differentiation.

### Sanger sequencing

To screen cell lines for the presence or absence of the specified AD-related gene variants, genomic DNA was isolated from each of the cell lines using the QuickExtract DNA extraction solution (ThermoFisher) and the genomic locus was amplified by PCR using the following primers: PSEN1, forward-CTAATGTTTGGGAGCCATCAC, reverse-GTTATGGGATGTACACGTTACC; PSEN2, forward-AGCAGGTCCAGAATCACTCAAG, reverse-TGGGAGACAGGATGGGGTG, and ApoE, forward-AGACGCGGGCACGGCTGT, reverse-CTCGCGGATGGCGCTGAG. PCR products were enzymatically purified using Cytiva ExoProStar PCR and Sequence Reaction Clean-up Kit (Fisher Scientific) then screened by Sanger sequencing (GeneWiz) to identify specific AD gene variants, using the primer sequence of GAGGAAAGAAAACACTCCAG for the PSEN1 locus, either AATCACTCAAGGTGGGGAGC or AGACGGAGAGAAGCGTGG for the PSEN2 locus, and CGCTTCTGCAGGTCATC for the ApoE locus.

### Cryosectioning, immunostaining, and microscopy

Undifferentiated colonies of hPSCs as well as differentiated retinal organoids derived from all cell lines were fixed using 4% paraformaldehyde in PBS for 30 min, and then washed three times with 1X PBS. For retinal organoids, samples were then equilibrated in 20% sucrose followed by 30% sucrose solutions. They were then embedded in OCT and flash frozen followed by cryosectioning at 12 μM thickness. Immunocytochemistry for all samples was then performed by permeabilizing samples using 0.2% Triton X-100 for 10 min followed by 3 washes with 1X PBS. Samples were then blocked using 10% donkey serum for 1 h at room temperature, followed by incubation with the respective primary antibodies diluted in 5% donkey serum and 0.1% Triton X-100 overnight at 4 °C. Primary antibodies used are listed in Supplemental Table 1. The next day, samples were washed 3 × in PBS before incubation with corresponding secondary antibodies in 5% donkey serum and 0.1% Triton X-100 for 1 h at room temperature. Following 3 more washes in PBS, samples were mounted on slides for visualization either on a Leica DM5500 upright microscope or a Nikon AR1 confocal microscope.

### ELISA

Aβ40 and Aβ42 ELISA kits (Thermofisher Catalog no. KHB3481 and KHB3441, respectively) were used for measurement of the Aβ42:Aβ40 ratio. Conditioned medium was collected from 5-month-old retinal organoids grown for 48 h followed by 40X concentration of samples for the Aβ42 ELISA using the Pierce Protein Concentrator Kit (Life Technologies), following manufacturer’s instructions. The ratio of Aβ42:Aβ40 in each sample was normalized to the control average values of Aβ42:Aβ40 measured as a relative fold change.

### Immunoblotting

Cell lysates were collected using the Mammalian Protein extraction buffer (Thermofisher, Catalog no. 78501) from cultures of 5 month old retinal organoids, with the addition of a protease-phosphatase inhibitor cocktail. A BCA assay kit was used for the quantification of protein concentration, and 50 µg of protein was used for all experiments. Protein samples were prepared with 4X sample buffer plus DTT and incubated at 70 °C for 10 min, then loaded into 4–15% gradient gels. After transfer to nitrocellulose membranes using the BioRad Transblot Turbo system, blots were then blocked using 5% milk with Tris buffered saline with 0.1% Tween 20 (TBST). The blocking step was followed by addition of primary antibody in the same TBST solution overnight at 4 °C. The next day, blots were washed again with TBST solution 3 × times for 5 min and incubated with secondary antibody for 1 h. Following the incubation step, the blots were washed with TBST solution. Imaging and quantification were performed using the Li-COR Odyssey CLx imaging system with protein expression normalized to loading controls. Images of full membrane blots are provided in Supplemental Figs. 1 and 2, with images showing full lanes for samples run.

### Transcriptional profiling of retinal organoids

Total RNA was extracted from retinal organoids using the Picopure RNA extraction kit following manufacturer’s instructions. The transcriptional profile of each sample was then analyzed using the Nanostring Alzheimer’s Disease panel (Nanostring Technologies Inc.) following manufacturer’s instructions and analyzed in nSolver Advanced analyses software. Briefly, 150 ng of each RNA sample was used for the characterization of transcriptional differences between populations using nSolver software, with gene expression standardized with 6 housekeeping genes. We used unadjusted *p* < 0.05 as a cutoff to determine the differentially expressed genes (DEGs). The Log2 of normalized gene expression of DEGs obtained from nSolver software (NanoString®) was input into *ClustVis* (https://biit.cs.ut.ee/clustvis/) to perform clustering analysis and generate the heatmap.Clustering used Euclidean distance and average linkage. Rows are centered, with unit variance scaling applied to rows. Both rows and columns are centered using correlation distance and average linkage. For pathway enrichment analysis, Gene Ontology terms for Biological Process using the *Enrichr* software (https://maayanlab.cloud/Enrichr/) was used based upon identified DEGs^70^. The Volcano plot was generated using GraphPad PRISM 9.5.0. Volcano plots represent the fold change (x-axis) and statistical significance level expressed as the − log10P value (y-axis).

### Statistical analyses

For studies comparing quantified differences between control and AD retinal organoids, a minimum of at least 3 biological replicates per cell line were used in each assay. Differences between control and AD retinal organoids were determined by student’s t-test analysis, with these analyses performed using GraphPad PRISM software version 9.5.0. *p* < 0.05 was considered statistically significant.

### Supplementary Information

Supplementary Information.

## Data Availability

All data generated or analyzed during this study are included in this published article [and its supplementary information files]. Raw data from the 12 sequencing reactions in Fig. 1 and nanostring transcriptional profiling in Fig. 4 has been deposited on the NCBI BioSample repository (https://www.ncbi.nlm.nih.gov/biosample/). Accession numbers for Sanger Sequencing in Fig. 1 is SAMN35074495-SAMN35074506, while accession numbers for Nanostring data in Fig. 4 is SAMN35159363-SAMN35159372.
