# Supplementary material for: Development of a three-dimensional organoid model to explore early retinal phenotypes associated with Alzheimer’s disease
Source: Sci Rep. 2023 Aug 24;13:13827. doi: 10.1038/s41598-023-40382-4 (PMC10449801; doi:10.1038/s41598-023-40382-4)
Supplement: Supplementary file 1 — Supplementary Information. [file 41598_2023_40382_MOESM1_ESM.pdf]

Supplemental Table 1: List of antibodies used in the study.

Antibody	Type	Source	Catalog	Dilution
Actin	Mouse monoclonal	Sigma	A5441	1:10,000
AT-8 (pTau)	Mouse monoclonal	Life Technologies	MN1020	1:100
BRN3	Goat polyclonal	Santa Cruz Biotechnology	SC-6026	1:200
CHX10	Mouse monoclonal	Santa Cruz Biotechnology	SC-365519	1:200
CRX	Mouse monoclonal	Abnova	H00001496-M02	1:100
GRIA3	Rabbit polyclonal	Cell Signaling Technology	4676T	1:500
MAP2	Rabbit polyclonal	Synaptic Systems	188011	1:200
NRL	Rabbit polyclonal	R&D Systems	AF2945	1:200
OCT4	Rabbit polyclonal	Stemgent	09-0023	1:200
SOX2	Goat polyclonal	R&D Systems	AF2018	1:1000
Tau (total)	Rabbit polyclonal	Dako	A0024	1:10,000
TRA-1-60	Mouse monoclonal	Stemgent	09-0010	1:1000
Tra-1-81	Mouse monoclonal	Stemgent	09-0011	1:1000
SNAP25	Mouse monoclonal	Millipore	MAP331	1:100
Synapsin-2	Rabbit polyclonal	Cell Signaling Technology	85852S	1:500
Synaptophysin	Rabbit polyclonal	Cell Signaling Technology	36406T	1:500
Synaptotagmin-1	Rabbit polyclonal	Cell Signaling Technology	14558S	1:500

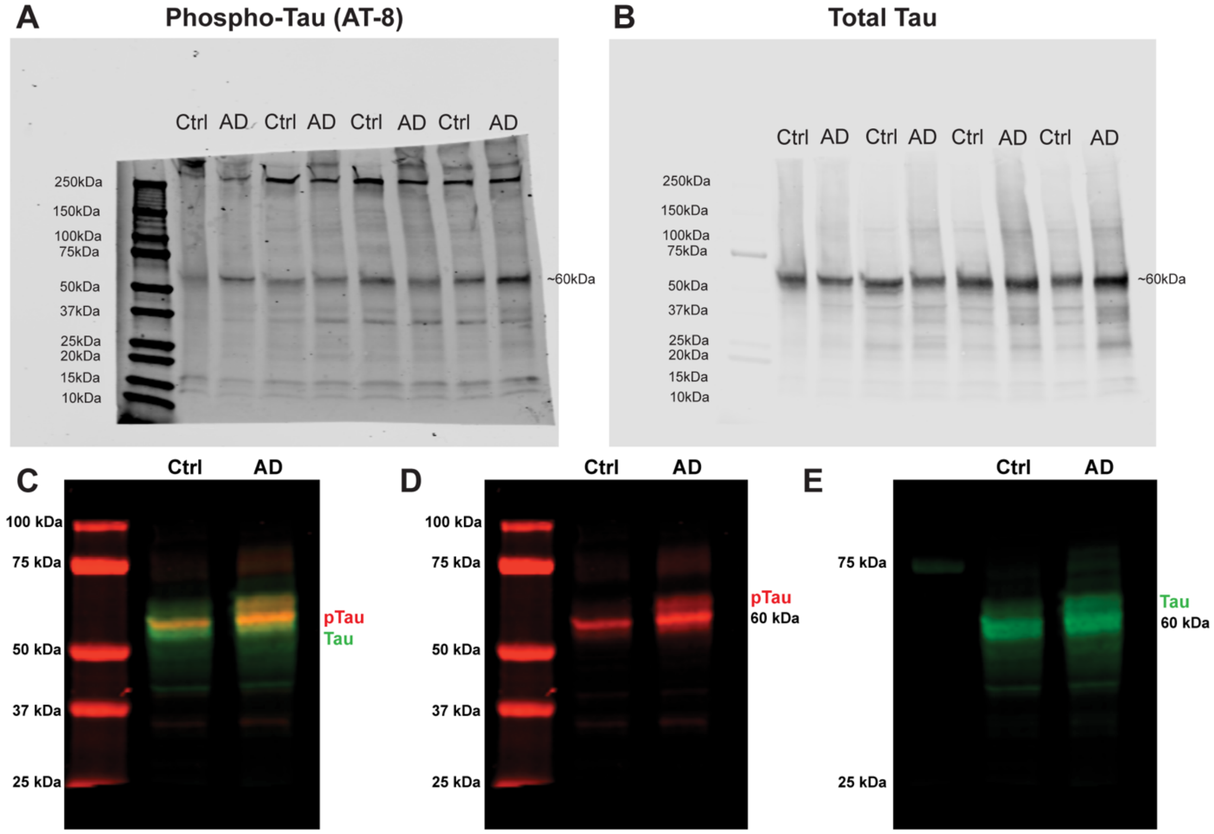

Supplemental Figure 1. Full gel images related to Figure 3. **(A-B)** Full blot images demonstrating the expression of phospho-Tau **(A)** and total Tau **(B)**, including full lanes and membranes. Note that membrane images in **A** and **B** are of the same membrane double-stained with respective antibodies and imaged in the red **(A)** and green **(B)** channels to ensure accuracy of quantification of protein samples. **(C-E)** Representative images from control and AD samples run on the same membrane and imaged in separate channels, as described for **A-B**.

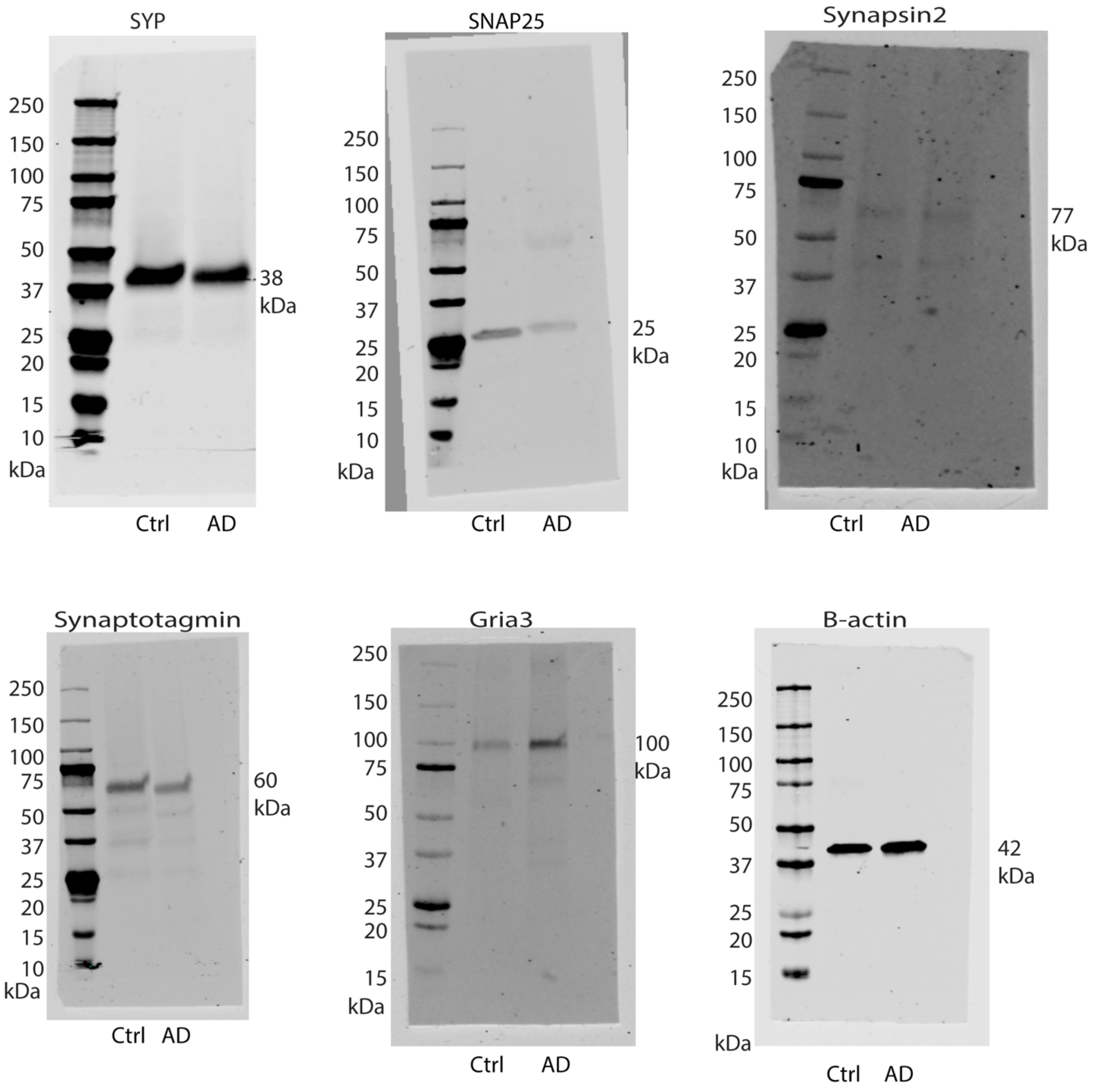

Supplemental Figure 2. Full gel images related to Figure 4 demonstrating full lanes and membranes to ensure specificity of the antibodies with bands at expected sizes.
